# Nasal septum changes in adolescent patients treated with rapid maxillary expansion

**DOI:** 10.1590/2177-6709.21.1.047-053.oar

**Published:** 2016

**Authors:** Tehnia Aziz, Francis Carter Wheatley, Kal Ansari, Manuel Lagravere, Michael Major, Carlos Flores-Mir

**Affiliations:** 1 Private practice, Edmonton, Alberta, Canada; 2 Graduate student in Computer Sciences, Boston University, Boston, Massachusetts, USA; 3 Assistant professor, University of Alberta, Department of Surgery, Edmonton, Alberta, Canada; 4 Assistant professor, University of Alberta, Department of Dentistry, Edmonton, Alberta, Canada; 5 Clinical assistant professor, University of Alberta, Department of Dentistry, Edmonton, Alberta, Canada; 6 Professor, University of Alberta, Department of Dentistry, Edmonton, Alberta, Canada

**Keywords:** Nasal septum, CBCT, Rapid maxillary expansion, Rapid palatal expansion

## Abstract

**Objective:**

To analyze cone-beam computed tomography (CBCT) scans to measure changes in nasal septal deviation (NSD) after rapid maxillary expansion (RME) treatment in adolescent patients.

**Methods:**

This retrospective study involved 33 patients presenting with moderate to severe nasal septum deviation as an incidental finding. Out of these 33 patients, 26 were treated for transverse maxillary constriction with RME and seven, who did not undergo RME treatment, were included in the study as control group. CBCT scans were taken before appliance insertion and after appliance removal. These images were analyzed to measure changes in nasal septum deviation (NSD). Analysis of variance for repeated measures (ANOVA) was used.

**Results:**

No significant changes were identified in NSD regardless of the application or not of RME treatment and irrespective of the baseline deviation degree.

**Conclusion:**

This study did not provide strong evidence to suggest that RME treatment has any effect on NSD in adolescent patients; however, the results should be interpreted with caution, due to the small sample size and large variation amongst individual patient characteristics.

## Abstract (pt)

**Objetivo:**

analisar imagens de tomografia computadorizada de feixe cônico (TCFC) para mensurar as alterações no desvio de septo nasal (DSN) após o tratamento com expansão rápida da maxila (ERM) em pacientes adolescentes.

**Métodos:**

o presente estudo retrospectivo incluiu 33 pacientes com desvio de septo nasal de moderado a severo, diagnosticado como um achado incidental. Dos 33 pacientes analisados, 26 tiveram a constrição maxilar transversal tratada por meio de ERM; 7 pacientes não foram submetidos à ERM, sendo incluídos no estudo como grupo controle. As imagens de TCFC foram obtidas antes da instalação do aparelho e após sua remoção, sendo analisadas para mensurar as alterações no DSN. A análise de variância para medidas repetidas (ANOVA) foi empregada.

**Resultados:**

não foram identificadas alterações significativas no DSN, independentemente da realização ou não do tratamento com ERM e do grau inicial de desvio.

**Conclusão:**

esse estudo não fornece evidências suficientes para sugerir que o tratamento com ERM produza qualquer efeito sobre o DSN em pacientes adolescentes. Porém, esses resultados devem ser interpretados com cautela, em virtude do tamanho reduzido da amostra e da grande variação das características individuais dos pacientes.

## INTRODUCTION

The reciprocal effects of nasal breathing on craniofacial development have been intensively investigated in the literature. According to Moss' functional theory, nasal respiration enables normal growth and development of the craniofacial structures.^1^ Moss hypothesized that undisturbed nasal airflow is a continuous stimulus for lowering the palate and for lateral maxillary growth, thereby indicating a close relationship between nasal breathing and dentofacial morphology. 

Nasal septal deviation (NSD) is defined as deviation of either the bony or cartilaginous septum or both from the facial midline. In humans, it has been hypothesized that significant nasal obstruction caused by NSD can affect nasal airflow and increase nasal airway resistance.^2^ Resultant impaired nasal breathing can lead to preferential mouth breathing which, if chronic, may cause craniofacial alterations. These potential changes consist of a long face syndrome characterized by narrow maxilla, steep mandibular plane, retrognathic mandible, increased lower facial height, lip incompetence, constricted alar bases and typically malocclusion consisting of a posterior crossbite.^2^

Rapid maxillary expansion (RME) is routinely used in Orthodontics to treat transverse maxillary constriction, posterior dental crossbite and crowding.^3^ Considering that maxillary bones form the periphery of the nasal cavity, it has been proposed that opening the mid palatal suture through RME could also result in lateral displacement of the nasal walls, thereby increasing nasal cavity dimensions.^4^

The earliest report of RME having an effect on nasal septal changes came in 1975 when it was discovered that RME treatment in a patient cohort also appeared to improve NSD.^5^ More recently, a 94% reduction in septal deviation was reported in children aged 5-9 years old, presenting with transverse maxillary constriction treated with RME.^6^ NSD correction was noted in the lower and middle half of the nasal cavity when compared to a nonexpansion control group. 

Although several studies have investigated the effect of RME on nasal cavity size and airway,^5^^,^ 7 ^-^ 11 there is a paucity of research on the changes caused by RME in the nasal septum. To our knowledge, only three studies have conducted a two-dimensional cephalometric analysis^5^^,^ 6 ^,^ 12 to assess nasal septal changes produced during RME. All of them used coronal views from posterior anterior radiographs. Two studies reported favorable improvement of septal deviation after RME^5^^,^ 6 treatment in growing patients, while one^12^ study reported no change in nongrowing patients aged 15-19 years old. However, these studies had some major limitations. There was lack of standardization in study design and the nasal septum was measured at one single radiographic image instead of its entirety by assessing different points along the septum. It was also unclear whether both pre- and postexpansion radiographs measured the septal change at a set landmark. Although improvement in nasal septum was reported after expansion, it was not clear whether the change was the same at each anatomical location along the nasal septum. 

Considering the importance of nasal breathing for the development of craniofacial structures, it would be beneficial to ascertain whether RME can reliably improve NSD and hence its detrimental effects on nasal breathing. Therefore, the objective of this study is to analyze three-dimensional changes of the nasal septum resulting from maxillary expansion in an adolescent patient sample. The use of three-dimensional imaging should overcome some of the limitations observed in previously conducted research.

## MATERIAL AND METHODS

This retrospective study fulfilled all ethical requirements and was approved by the Health Research Ethics Board at the University of Alberta.

Patient samples were obtained from a previously conducted randomized clinical trial^14^ at the Department of Dentistry at University of Alberta during an 18-month period. A total of 33 patients with varying degrees of NSD at T_1_ (prior to rapid maxillary expansion [RME] treatment) were selected from an available pool of CBCT scans of 120 patients through a brief visual inspection of the entire nasal septum of each patient. Patients with nasal septum deviation were identified from transverse and coronal views of cone-beam computed tomographic (CBCT) records taken prior to treatment with RME (or without RME for control patients). 

Based on a previous publication,^13^ septal deviation was considered moderate to severe (clinically meaningful deviation) if the deflection of the nasal septum from the mid-sagittal plane was greater than 9 degrees, and mild (not clinically meaningful deviation) if deviation was less than or equal to 8 degrees in any isolated CBCT scan. 

The final sample consisted of:

» 14 patients treated with RME with moderate to severe NSD at T_1_ (more than 9 degrees);
» 12 patients treated with RME with mild NSD at T_1_ (less than 9 degrees);
» 7 untreated patients with RME with moderate to severe NSD at T_1_ (control group).

The BAME (bone anchored maxillary expansion) sample had a mean age of 14.2 ± 1.3 years, the TAME (tooth anchored maxillary expansion) sample had a mean age of 14.1 ± 1.4 years and finally the control sample had a mean age of 12.9 ± 1.2 years. Individual matching was not possible due to unequal sample sizes. 

RME was carried out until posterior dental crossbite overcorrection by 20% was achieved (maxillary lingual cusps overlapping with lingual inclines of mandibular buccal cusps). After active expansion treatment, the screw was fixated with a composite resin into the turn-key mechanism of the appliance. The appliance was retained for a total of six months from the time of insertion. CBCT scans taken at T_1_ (at baseline, before expansion) and T_2_ (after appliance removal) were analyzed for this study. (For more detailed information on the methods of the previously conducted randomized trial please refer to reference^14^). 

All CBCT scans were taken with either a NewTom (18 patients) or an i-CAT (15 patients). Images were converted into DICOM format software with a voxel size of 0.25 mm. All images at T_1_ and T_2_ for each patient were then uploaded to OsiriX DICOM Viewer (v. 5.8, 32 bit, Pixmeo, Geneva, Switzerland). 

**Figure 1:**
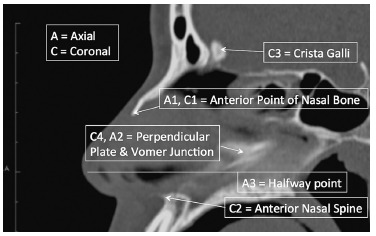
- Description and location of landmarks in sagittal view for axial and coronal image generation.

Based on a previous publication,^15^ the following steps were followed:

Landmarks were identified in the 3-D viewer/2-D orthogonal MPR mode in OsiriX for each patient in sagittal view (Table 1, Fig 1). 

**Table 1:** - Descriptions of landmarks in sagittal view for image generation.

**Axial (A) and coronal (C) landmarks**	**Anatomical location**
A1	Most anterior point of nasal bone (axial view).
A2	Point that depicts the junction of perpendicular plate of ethmoid bone and vomer (axial view).
A3	Midway point between A2 (C4) and C2. Anatomically found between the anterior nasal spine and vomer/perpendicular plate of ethmoid junction in vertical direction (axial view).
C1	Anterior point of nasal bone (coronal view)
C2	Most anterior point of anterior nasal spine (coronal view).
C3	Mid point of crista galli (coronal view).
C4	Junction of perpendicular plate of ethmoid bone and vomer (coronal view).

Although A2 and C4 are the same landmarks in sagittal view, on A2 slice, nasal septum is measured in anterior to posterior. On C4 slice, it is measured from to inferior to superior view.

Based on the landmarks identified on sagittal view, three axial (A1, A2, A3) and four coronal DICOM landmarks (C1, C2, C3, C4) for each patient at each time point were isolated (Fig 1). 

These landmarks were: the most anterior point of nasal bone (A1, C1), perpendicular plate and vomer junction (A2, C4), anterior nasal spine (C2), crista galli (C3), halfway point between anterior nasal spine and perpendicular plate/vomer junction (A3).

The landmarks were chosen due to their ease of identification based on anatomical locations and because they could reasonably cover the boundaries of normal septal anatomy in anterior-posterior and inferior to superior directions. Landmark A3 was the only landmark not identified by an anatomical structure.^15^ A total of 14 images for every patient was analyzed considering T_1_ and T_2_. These images were then transferred to MATLAB (MathWorks R2013b, Natick, Massachusetts) for NSD analysis. One researcher registered the time point (T_1_ or T_2_ DICOM image) in the MATLAB software, but was blinded to the degree of septal deviation or whether it was a treatment or a control patient. This software enabled the septum to be systematically traced and analyzed. 

During analysis in MATLAB, the axial images (images A1, A2, A3) were traced from anterior to posterior direction. For example, for axial image A1, the nasal septum was systematically traced by placing points approximately 1-2 mm apart along its anterior posterior course. Similarly, in coronal images (C1, C2, C3, C4), the nasal septum was traced in entirety from superior to inferior direction by placing points 1-2 mm apart. 

The data from NSD measurements from MATLAB software were automatically transferred to a comma separated value (csv) spreadsheet. Once data analysis was complete, data were further copied to an Excel spreadsheet for ease of statistical analysis with SPSS program.

**Figure 2:**
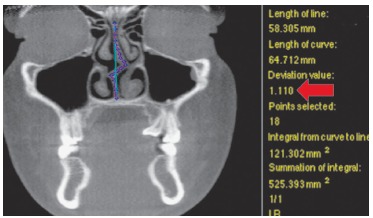
- MATLAB analysis for degree of tortuosity.

For the present study, NSD was quantified based on the "degree of tortuosity" or the ratio of length of the curve to the length of an imaginary line in the mid sagittal plane (Fig 2 - red arrow points to ratio). In other words, the degree of tortuosity is an absolute measurement of the degree of septal deviation from the midline at each identified landmark in both coronal and/or axial views.

## STATISTICAL TESTS

### Reliability and measurement error

Intrarater reliability and measurement error were conducted for identification of landmarks in sagittal view in OsiriX and then as NSD tracing in MATLAB (from the selected DICOMs based on landmark identification/image isolation in OsiriX). All measurements were repeated three times with at least five days apart. 

Landmark identification was done in OsiriX with X, Y and Z coordinates noted for anterior point of the nasal bone (landmarks A1/C1), vomer and perpendicular plate junction (landmarks A2/C4), anterior nasal spine (C2), crista galli (landmark C3), and halfway point between anterior nasal spine and vomer/perpendicular plate junction (A3). Nasal septum tracing and NSD measurement ratio in MATLAB for each image (A1, A2, A3, C1, C2, C3 and C4) were also recorded. 

Intraclass correlation with consistency under two-way mixed model was tabulated in SPSS for both landmark identification and nasal septum measurements. 

### Main study statistical tests 

Statistical analysis was carried out with the aid of SPSS (version 21) using alpha = 0.05. Analysis of variance for repeated measures (ANOVA) was performed with two within-subject factors and one between-subject factor. Baseline septal deviation of "mild" and "moderate to severe" was considered between subjects factor. Time (T_1_ and T_3_, two levels) and landmark (A1, A2, A3, C1, C2, C3 and C4, 7 levels) were the two within-subject factors. 

## RESULTS

The intraclass correlation coefficients and corresponding confidence interval (95%) for both OsiriX landmark identification and MATLAB NSD measurements are listed in Tables 2 and 3. Location of most landmarks indicated good agreement^16^ between parameters by high ICC values (> 0.8). Minimum, maximum and mean measurement error for both OsiriX landmark identification and MATLAB NSD ratios are listed in Tables 4 and 5. Mean measurement error in OsiriX was in the range of 0.5-2.2 mm with A3 having the largest error in all coordinates (1.96 to 2.2 mm). Difference between landmark coordinates in OsiriX measured at three different time points for reliability was not greater than 4 mm. MATLAB NSD ratios at all landmarks were less than 0.02. 

**Table 2:** - Intrarater reliability for OsiriX landmark/image identification.

**Landmark/image**	**X coordinate**	**Y coordinate**	**Z-coordinate**
A1/C1	0.980 (0.943 - 0.995)	0.974 (0.925 - 0.993)	0.994 (0.983 - 0.998)
A2/C4	0.963 (0.897 - 0.990)	0.974 (0.926 - 0.993)	0.986 (0.959 - 0.996)
A3	0.941 (0.839 - 0.984)	0.929 (0.810 - 0.980)	0.988 (0.965 - 0.997)
C2	0.980 (0.943 - 0.995)	0.978 (0.937 - 0.994)	0.990 (0.971 - 0.997)
C3	0.998 (0.993 - 0.999)	0.973 (0.924 - 0.993)	0.986 (0.959 - 0.996)

**Table 3:** - Intrarater reliability for MATLAB NSD measurements.

**Image**	**ICC**	**Confidence interval**
A1	0.948	(0.871 - 0.983)
A2	0.993	(0.982 - 0.998)
A3	0.872	(0.702 - 0.957)
C1	0.947	(0.867 - 0.983)
C2	0.914	(0.791 - 0.972)
C3	0.904	(0.771 - 0.969)
C4	0.941	(0.854 - 0.981)

**Table 4:** - Measurement error in millimeters for OsiriX landmark/image identification.

**Landmark/image**	**X coordinate**	**Y coordinate**	**Z coordinate**
A1/C1	1.11 (0 - 2.24)	1.23 (0.38 - 2.77)	1.23 (0.41 - 2.67)
A2/C4	1.32 (0.3 - 3.64)	1.44 (0.37 - 2.73)	1.78 (0.09 - 3.43)
A3	1.96 (0.67 - 3.71)	2.2 (1.25 - 3.27)	2.03 (0.48 - 3.49)
C2	1.58 (0.56 - 3.25)	1.52 (0.23 - 3.14)	1.61 (0.13 - 3.65)
C3	0.52 (0.28 - 0.84)	1.89 (0.59 - 3.51)	1.64 (0.41 - 3.61)

**Table 5:** - Measurement error for MATLAB NSD ratio..

**Image**	**Mean ratio**
A1	0.0016
A2	0.0042
A3	0.0069
C1	0.0025
C2	0.0023
C3	0.0166
C4	0.0090

There was no significant difference in NSD according to time [F (1.24) = 0.2, *p* = 0.659]. There was also lack of evidence for differences in NSD at images A1, A2, A3, C1, C2, C3, C4 with time (time*landmark location) [F (2.93, 70.24) = 0.205, *p* = 0.889]. 

Partial eta square was 0.008 for time*spatial image accounting for only 0.8 % of variance explained by the effect of RME on NSD. Baseline septal deviation of mild or moderate to severe had no effect change at spatial landmarks with time (baseline deviation*spatial image*time) [F (2.93, 70.24) = 1.85, *p* = 0.147, accounting for only 7% of variance in NSD]. 

## DISCUSSION

The purpose of this retrospective study was to investigate the effect of RME treatment on patients presenting with nasal septal deviation. RME alone was conducted in these patients for the treatment of transverse maxillary deficiency, whereby NSD was discovered as an incidental finding on their CBCT scans prior to treatment. 

There is no "gold standard" test to diagnose septal deviation^17^ and different protocols for measuring septal deviation have been identified in the literature. The degree of tortuosity measurement was used in this study to compare the length of the curve of the deviated septum to the length of an ideal straight septum. This measurement solely measured the nasal septum in isolation and did not classify or include other confounding nasal pathology that could be the reason for septal deviation, such as turbinate hypertrophy or mucosal swelling. Therefore, this measurement method was well suited to the objective of our study. 

Landmark identification in OsiriX and NSD ratios in MATLAB were indicative of good reliability. It was ascertained that identifying the location of landmark A3 with certainty was challenging. It was the only landmark that was not associated with a hard tissue anatomical structure, and rough approximation in space was made on all DICOMS without a ruler to accurately measure the half way point between anterior nasal spine and the vomer, and perpendicular plate junction. Although reliability at A3 for both OsiriX and MATLAB was suggestive of good reliability, a mean measurement error close to 4 mm was reported in x, y and z coordinates. 

Owing to the retrospective nature of the study and the available CBCT records of patients that have undergone RME, sample size was less than ideal. Nevertheless, our findings were similar to a recently conducted study^12^ in two dimensions, whereby no change in nasal septal deviation was identified pre- and postmaxillary expansion. On the other hand, this study was different than the previous one,^12^ since the analysis of septal deviation was based on three-dimensional measurements on CBCT as opposed to two-dimensional on a posterior-anterior cephalogram. To date, this study appears to be the only one comparing the effects of RME on NSD using three-dimensional analysis of CBCT scans. 

The main finding of this investigation was that rapid maxillary expansion had no effect on patients that had nasal septal deviation at baseline, as measured at images in axial and coronal views. Furthermore, mild or severe baseline deviation had no statistically significant effect on NSD change, as measured at set landmarks. The time difference between T_1_ and T_2_ in the treatment group was similar to that of the control group; neither one of the groups had statistically significant changes in NSD over a 6-month period. It could be challenging to identify the true effect of RME treatment on NSD due to relatively small sample size and individual patient variation. In fact, four patients out of a sample of 26 (15%) depicted subjective visual improvement, as determined by one author in NSD from RME at mostly the coronal location of C3 (one at A3) (Figs 6 to 9). All four presented with baseline deviation of moderate to severe, but it is unclear as to why others with severe deviation and similar characteristics did not have a similar change. This is parallel to the conclusions of Harvolds primate study^18^ whereby, even though the experiment protocol and sample characteristics were standardized, the animals responded and adapted to nasal obstruction quite differently. In fact, based on the statistical model, only 7% of variance in NSD could be attributed to RME treatment.

It has been proposed^19^ that early intervention with RME (i.e., before palatal suture starts closing) in prepubescence would result in greater skeletal than dental change. Given that all patients in this study were adolescents, it is possible that lack of statistically significant change in NSD was the result of subjects having more advanced craniofacial development. In addition, increased bone density (calcification) of surrounding craniofacial structures in adolescence can offer greater resistance to skeletal change from RME. In contrast, in patients with mixed and deciduous dentition, studies^20^^,^ 21 have reported the effect of RME to be attributed to between one half to two-thirds skeletal change. In fact, the studies^5^^,^ 6 that reported favorable effects of RME on NSD consisted of patients recruited prior to their adolescent growth spurt. 

Although there is a lack of studies examining the effect of RME on NSD, there are several studies^5^^,^ 7 ^-^ 10 investigating the influence of RME treatment on nasal airway. However, there still lies a great deal of ambiguity in the literature with respect to nasal airway changes from RME due to conflicting findings. This ambiguity could be attributed due to different expansion protocols, different measurement methods to assess nasal airway change, patients with varying degrees of skeletal maturation, individual patient variation with or without concurrent pathologies, such as infections and allergies causing mucosal edema. 

## LIMITATIONS

This study did not provide strong evidence to suggest that RME treatment has any effect on NSD in adolescent patients, but the results should be interpreted with caution, due to the small sample size and large variation amongst individual patient characteristics. In this sense, this could be considered a pilot study testing this novel methodology.

Although potential differences between bone- or tooth-anchoraged expansion appliances over NSD could not be considered because of the limited number of subjects per group, the reality is that the expansion anchorage site may be of little real impact, as stated in the previous RCT^14^ using the larger sample of 120 subjects in which dentoalveolar and skeletal changes were pretty similar regardless of expansion anchorage. 

## CONCLUSIONS

This study did not provide strong evidence to suggest that RME treatment has any effect on NSD in adolescent patients; however, the results should be interpreted with caution, due to the small sample size and large variation amongst individual patient characteristics. 
